# Carbuncles Successfully Treated with Carbolic Acid and Salicylic Acid Injections

**Published:** 1887-10

**Authors:** C. H. Wilkinson

**Affiliations:** Surgeon in charge of St. Mary’s Hospital, Galveston, Texas


### CARBUNCLE SUCCESSFULLY TREATED WITH CARBOLIC ACID AND SALICYLIC ACID INJECTIONS.

BY C. H. WILKINSON, M. D.,
(Surgeon in charge of St. Mary's Hospital, Galveston, Texas.)

On December 26, 1886, Clarance Baxter, æt. 33, tottered into my office, and threw himself, or, rather, fell, into a chair, exclaiming in a feeble voice that he had a carbuncle and desired me to treat it. The diseased process extended from the "inferior occipital ridge" to the fifth cervical vertebra, and nearly from ear to ear, and had then existed for fourteen days. The integument included in the space just mentioned was perforated by fifteen or twenty minute sinuses, through which a thin, yellowish, offensive pus, or rather ichor, was exuding at the rate of several ounces daily, while so tender and painful were the parts involved that opiates had to be taken in order to secure any repose whatever, day or night.

Mr. Baxter's general condition was very much below a healthy standard. He was pale, feverish, nervous and haggard; all the result of the terrible affliction he was carrying around on the back of his neck, and had been sent to me by his former medical attendant in order to receive the benefit of the carbolic acid treatment. The disease, however, had progressed too far to receive the abortive plan of treatment, i. e, injection of pure concentrated carbolic acid into the most prominent sinuses, so I directed the following wash to be used, diluting it from time to time as occasion required:

| | | |
|---|---|---|
| ℞. | Acid, carbolici | ℥ i, |
| | Acid, salicylici | ℥ i, |
| | Soda boracis | ʒ i, |
| | Glycerinæ | ℥ i, |
| | Spt. rectificat | ℥ i, |
| | Olei sassafras | ʒ i, |
| | Aquæ puræ, ad | ℈ i. M. |

Selecting an ounce syringeful of the undiluted mixture, I injected the same through some two or three of the most prominent sinuses, *and out in the direction of the most indurated portions of the carbuncle*, while a weaker portion was injected into the center of the diseased tissues in order to clean and stimulate that portion of the ulcer. No pain was complained of by the patient, from the use of this application, and having cleared away all exudation of pus and shreds of necrosed cellular tissue, I applied absorbent cotton and a well-fitting roller bandage around the neck and forehead, and directed him to call again on the next day.

The preceding plan of treatment was pursued daily thereafter for about a week, when it was found that the disease was fairly under control, and that the stronger washes could be dispensed with. Weaker solutions—1 part to 6 of water—were used, however, for two or three weeks longer, merely in order to clear away debris, and to stimulate healthy granulations.

A tonic of quinine and iron was given thrice daily, the patient directed to eat well and exercise in the open air, and the washes were kept up until the 10th day of March, 1887, when Mr. B. was discharged, well and hearty, and full of thanks for the happy termination of so dreadful a malady.

A recital of the foregoing case, with the minutæ observed in its management, is given for the purpose of demonstrating how simple a process may be relied upon in conquering a disorder so terrible as advanced carbuncle. So broken down was this patient when first observed that a question of doubt arose in my mind as to the power of any remedy to arrest his disease; yet, without pain, and in a few days, his carbuncle is brought under subjection by a remedy both safe, reliable and unrevolting, an indorsement which no other plan of treatment known to the profession can be said to enjoy at present.

Carbolic acid, whether diluted or in full strength, certainly exercises a prompt, if not a specific, influence over carbuncular inflammation. It has been my fortune to watch the action of this most excellent remedy in these and kindred ailments for eight or ten years past, and I can confidently assert that no death should ever occur from carbuncle where carbolic acid has been employed *in time*, either in the manner set forth by me in the November, 1885, number of *Daniel's Texas Medical Journal*, or in a more diluted form as explained by me in the present article. The addition of the salicylic acid and other ingredients is made for the purpose of simply aiding the phenol in its work of stimulating granulations, deodorizing, etc.

The mixture referred to herein is a most valuable one, not only for carbuncle, but for many other surgical ailments, and I have employed it for several years in the wards of St. Mary's Hospital with the very highest satisfaction. In concluding these lines upon the action of carbolic acid on carbuncle, I desire to disclaim priority in the application of this drug to the malady under consideration, knowing full well that he who attempts priority in anything in the practice of medicine, undertakes a hurclean task that has rarely ever been successfully accomplished by any one; mummies having been heard to exclaim: "I used that remedy myself, in the days of Ptolemy."

I simply desire to attract the attention of the profession to an improved method of dealing with a terrible disease—a disease that often kills, and when it spares the life of its victim, seldom fails to leave behind the memory of a torture that lasts forever.—*Texas Courier Record of Medicine.*

